# Supplementary material for: Spatiotemporal patterns and prediction of landscape ecological security in Xishuangbanna from 1996–2030
Source: PLoS One. 2023 Nov 8;18(11):e0292875. doi: 10.1371/journal.pone.0292875 (PMC10631692; doi:10.1371/journal.pone.0292875)
Supplement: Attachment — Submitted filename: renamed_e7d8d.docx [file pone.0292875.s001.docx]

In this study, ‘transboundary eco-security’ was selected as the entry point for the study.

Based on the change of land use in Xishuangbanna, with the help of "3S" technology, landscape ecology theory, and gray prediction model, the spatial and developmental trends of landscape ecological security in Xishuangbanna from 1996-2030 could be determined.

The language of the whole essay is fluent, and the selected topic has certain research significance, but there are many places where the elaboration is unclear and insufficient to explore.

1）In the section of 2.3.2, why are 8km/10km and 12km chosen as the study area evaluation scale for comparison? Why do terrain and slope serve as the basis for evaluation units in the study area? How was the comparison made?

2) In the results section, the current findings can be further analyzed. For example, is there a spatial and temporal correlation of ecological safety patterns? How does the distribution of landscape ecological safety patterns vary from year to year for different grades?

3) The discussion section should be further discussed in depth. It should corroborate the feasibility and accuracy of the results of the research through previous studies, rather than just stating the importance of this study. In addition, the discussion in this paper is not deep enough and should be expanded according to the contents.
