# Supplementary material for: Spatiotemporal patterns and prediction of landscape ecological security in Xishuangbanna from 1996–2030
Source: PLoS One. 2023 Nov 8;18(11):e0292875. doi: 10.1371/journal.pone.0292875 (PMC10631692; doi:10.1371/journal.pone.0292875)
Supplement: Attachment — Submitted filename: Response to Reviewers.docx [file pone.0292875.s002.docx]

Dear Professors and editors,

Thank you very much for processing our manuscript (PONE-D-23-15562) entitled " *Spatiotemporal evolution and trend prediction of the Xishuangbanna landscape ecological security pattern from 1996-2030*", by Zhuoya Zhang, Hailong Ge, Xiaona Li, Xiaoyuan Huang, Siling Ma and Qinfei Bai. We highly appreciate your comments and revision suggestions. All the comments from the reviewers are constructive and valuable. We changed the title to "*Spatiotemporal patterns and prediction of landscape ecological security in Xishuangbanna from 1996-2030*" by integrating the reviewers' comments and suggestions. Our point-to-point responses and corrections are displayed with blue fonts in the following text. We have made major and careful revisions closely following the comments. Thus, the quality and clarity of our manuscript are improved. We sincerely hope our responses are satisfactory.

If you have any question, please let us know.

Yours sincerely,

Xiaoyuan Huang

Associate Professor

Faculty of Geography and Ecotourism

Southwest Forestry University

Kunming, China, 650224

E-mail: [hxy19792721@163.com](mailto:hxy19792721@163.com)

**-Response to Reviewer 1**

Thank you very much for your time involved in reviewing the manuscript and your very encouraging comments on the merits.

**1. In the section of 2.3.2, why are 8km/10km and 12km chosen as the study area evaluation scale for comparison? Why do terrain and slope serve as the basis for evaluation units in the study area? How was the comparison made?**

Answer: Thank you for pointing out our problem. We apologize for not expressing our thoughts clearly. We acknowledge that there were issues with our expression. The selection of evaluation units in the study area is crucial for ecological safety evaluation. We have chosen the evaluation units based on the extent of the study area and the patch area of different landscape types. Additionally, we have appropriately cited the references used in this process. Specific modifications and improvements are as follows:

(1) Determination of the evaluation unit of the study area

Through the Fishnet Analysis tool in ArcGIS 10.8, taking into account the study area’s extent and the patch areas of different landscape types, following the 2-5 times principle based on patch size[42-44], the study area is divided into 243 square grid cells of 10×10 km each using an equidistant method. A risk index is then constructed to determine the risk value at the center point of each landscape unit, which is utilized to calculate the landscape ecological security index (Fig. 3).

**2. In the results section, the current findings can be further analyzed. For example, is there a spatial and temporal correlation of ecological safety patterns? How does the distribution of landscape ecological safety patterns vary from year to year for different grades?**

Answer: We would like to express our gratitude to the reviewers for this comment, and in the results section, we have included a spatial autocorrelation analysis of ecological security in Xishuangbanna landscapes and a transfer analysis of ecological security. The spatial autocorrelation analysis enables a comprehensive examination of the spatial correlation of ecological security patterns. Additionally, the transfer analysis facilitates the study of inter-annual distributional changes in the ecological security patterns of landscapes at various levels. The new chapter titles are Spatial autocorrelation analysis, Analysis of landscape ecological security transfer and Spatial autocorrelation of landscape ecological security.

**3. The discussion section should be further discussed in depth. It should corroborate the feasibility and accuracy of the results of the research through previous studies, rather than just stating the importance of this study. In addition, the discussion in this paper is not deep enough and should be expanded according to the contents.**

Answer: We appreciate you pointing that out. We have revised the discussion section to cover the following topics: Ecological Security Index and ecological security, Tempo-spatial changes of landscape ecological security, Contributions and limitations. The feasibility and accuracy of our findings were demonstrated through comparison with other experiments. Furthermore, the causes of changes in landscape ecological security were further discussed.

We would like to express our gratitude for the time you have dedicated to reviewing our manuscript and providing us with this valuable opportunity for improvement. Your input has been immensely helpful, and we have taken it into careful consideration during the revision process. We sincerely hope that you will find the revised version to meet your expectations.

**-Response to Reviewer 2**

We deeply appreciate the time you invested in reviewing our manuscript and the kind words of encouragement regarding its merits. Your thoughtful evaluation has been invaluable to us. Thank you sincerely for your dedication and support.

**1. The title of this article does not reflect the research contents well.**

Answer: Thank you very much for your valuable comments on our paper. We have reconsidered the title and revised it according to your guidance. The revised title is: *"Spatiotemporal patterns and prediction of landscape ecological security in Xishuangbanna from 1996-2030"*

**2. The English writing need improved for the requirement of the scientific paper.**

Answer: Thank you for your careful review and valuable comments. We have asked a professional touch-up agency to touch up the language of the article before submission, however, under your guidance, we found that part of the writing may not be expressed accurately. To ensure that the language of the article is of the best standard, we have carefully reviewed the whole article again and made further revisions to address the parts that may be unclear or prone to misinterpretation. We expect our revisions to be at the level of a scientific paper

**3. The introduction has no smooth transition between paragraphs, lacks a description of the current state of research in the relevant field, and has a high rate of rewriting.**

Answer: Thank you very much for your suggestions for revision. As per your request, I have revised the introduction to address the lack of flow between paragraphs and added a description of the current state of research in the relevant field. In addition, I have reduced the frequency of rewrites.

The problem of poor articulation has been resolved. I have ensured that paragraphs are more naturally connected to each other by incorporating transition words and sentences.

To address the lack of description of the current state of research in the relevant field, I have cited the latest research findings and published papers that provide cutting-edge advances in the field today.

Finally, I have also made an effort to minimize the frequency of rewrites in order to ensure coherence and consistency in the text.

**4. The location map of the study area should be added in the overview of the study area section.**

Answer: Thank you very much for your review comments. As per your suggestion, I have added a location map of the study area in Overview of the study area.

Fig. 1 location of the study area

The map images we use come from Environmental Sciences, Chinese Academy of Sciences (<http://www.resdc.cn/>)

**5. The error of formula in research method section should be corrected, such as formula 3.**

Answer: Thank you very much for your review comments. I sincerely apologize for my formula errors in the research methods section.We have carefully examined and corrected the formula errors in the Research Methods section, including fixing Equation 3. We will ensure that the correct mathematical notation and expressions are followed during the revision process, with proper validation and review. Here is our revised formula 3:

$$R_{i}=E_{i}.F_{i}$$

**6. Line 153 to line 157, what is the number of the “68 rubber forests”? And the title of picture 1 should be revised.**

Answer: Thank you very much for your review comments. We apologize for the ambiguity in our expression, we originally meant: 68 out of 305 random sample points for rubber forests, and we have revised lines 153-157 as follows:

In this study, a total of 305 GPS sampling points were distributed along the main roads in Xishuangbanna Prefecture. Among these points, there were 68 rubber plantation areas, 73 tea gardens, 85 forested areas, 23 developed lands, 16 water bodies, and 40 cultivated lands The total accuracy Kappa coefficients after phase 5 image classification were 85.9%, 86.7%, 89.9%, 93.5%, and 87.3%, respectively, which met the study needs.

Meanwhile, we have revised the title of picture 1, it is now:

**Fig. 2 Land use type changes in Xishuangbanna from 1996 to 2017**

**7. The size of assessment grid should be written as 8 km*8 km, and the related selection criteria you referred should be cited.**

Answer: Many thanks to the reviewer for your guidance. I apologize for the omission in the description of the dimensions of the evaluation grid and the associated selection criteria. Following your suggestion, I will explicitly state the size of the evaluation grid as 10 km × 10 km in the text, and this correction will more accurately reflect the actual size used in the study. Combining your and Reviewer #1's suggestions, we have rewritten this section, and we have selected the evaluation cells based on the extent of the study area and the size of the patches in different landscape types. Specific modifications and improvements are as follows:

(1) Determination of the evaluation unit of the study area

Through the Fishnet Analysis tool in ArcGIS 10.8, taking into account the study area’s extent and the patch areas of different landscape types, following the 2-5 times principle based on patch size[42-44], the study area is divided into 243 square grid cells of 10×10 km each using an equidistant method. A risk index is then constructed to determine the risk value at the center point of each landscape unit, which is utilized to calculate the landscape ecological security index (Fig. 3).

**8. In general, the description of the results is not clear enough, and the structure of this paper is unclear, such as section of 3.2 and 3.3 should be merged.**

Answer: Many thanks to the reviewers for reviewing our paper and providing feedback. We apologize for the lack of clarity in the description of the results and the structure of the paper.

We acknowledge the concerns raised by the reviewers regarding the clarity of the result descriptions and the structure of the paper. We will make additional revisions to the paper based on your suggestions to ensure that the descriptions of the results are clearer. First, we have merged sections 3.2 and 3.3, which is now titled Space and temporal analysis and prediction. Additionally, we have divided this section into different tertiary headings: Ecological Security Index Calculation and Prediction Model; Landscape ecological security analysis; Analysis of landscape ecological security Taking into account your suggestion and that of reviewer #1, we have also added spatial autocorrelation analysis of ecological security and transfer analysis to enhance the analysis of landscape ecological security.

**9. In the discussion part, the discussion is not profound enough, and the analysis of the results lacks references to support the conclusion. Conclusion should be refined.**

Answer: Thank you very much for pointing out our problems. Taking into account your suggestions with reviewer 1, we have restructured the discussion section into three parts: Ecological Security Index and ecological security; Tempo-spatial changes of landscape ecological security; Contributions and limitations. By comparing our findings with those of other experiments, we have demonstrated their feasibility and accuracy. In addition, the reasons for the changes in landscape ecological security are further explored. At the same time, we further refine the conclusions to ensure that they accurately reflect the findings and contributions of the study. We clearly articulate the significance and potential implications of the conclusions.

**10. The manuscript has more writing errors, which should be carefully checked by the author**

Answer: Thank you for the detailed review. We have carefully and thoroughly proofread the manuscript to correct all the grammar and typos.

We would like to take this opportunity to thank you for all your time involved and this great opportunity for us to improve the manuscript. We hope you will find this revised version satisfactory.
