# Supplementary material for: Spatiotemporal patterns and prediction of landscape ecological security in Xishuangbanna from 1996–2030
Source: PLoS One. 2023 Nov 8;18(11):e0292875. doi: 10.1371/journal.pone.0292875 (PMC10631692; doi:10.1371/journal.pone.0292875)
Supplement: Attachment — Submitted filename: renamed_35624.docx [file pone.0292875.s003.docx]

The author has done an excellent revision. I need to give appreciation to the author for the hard work done to improve the writing of the manuscript according to the reviewers' suggestions. There are some minor issues that need to be corrected, after which the manuscript can be considered approved for publication.

1）The title of line 222 is "Spatial analysis method", which is an inaccurate subheading. The methods in the text, such as spatial autocorrelation analysis, landscape index and landscape security pattern, are also spatial analysis methods. Consideration could be given to replacing them with a specific method.

2）There is a formatting error on line 174

3) Coordinates are missing in Figures 2, 3, 5, and 7
