# Supplementary material for: Spatiotemporal patterns and prediction of landscape ecological security in Xishuangbanna from 1996–2030
Source: PLoS One. 2023 Nov 8;18(11):e0292875. doi: 10.1371/journal.pone.0292875 (PMC10631692; doi:10.1371/journal.pone.0292875)
Supplement: Attachment — Submitted filename: Response to Reviewers.docx [file pone.0292875.s004.docx]

Dear Professors and editors,

Thank you very much for processing our manuscript (PONE-D-23-15562) entitled " *Spatiotemporal patterns and prediction of landscape ecological security in Xishuangbanna from 1996-2030*", by Zhuoya Zhang, Hailong Ge, Xiaona Li, Xiaoyuan Huang, Siling Ma and Qinfei Bai. We highly appreciate your comments and revision suggestions. All the comments from the reviewers are constructive and valuable. Our point-to-point responses and corrections are displayed with blue fonts in the following text. We have made major and careful revisions closely following the comments. Thus, the quality and clarity of our manuscript are improved. We sincerely hope our responses are satisfactory.

If you have any question, please let us know.

Yours sincerely,

Xiaoyuan Huang

Associate Professor

Faculty of Geography and Ecotourism

Southwest Forestry University

Kunming, China, 650224

E-mail: [hxy19792721@163.com](mailto:hxy19792721@163.com)

**-Response to Reviewer 1**

Thank you very much for your time involved in reviewing the manuscript and your very encouraging comments on the merits.

**1. The title of line 222 is "Spatial analysis method", which is an inaccurate subheading. The methods in the text, such as spatial autocorrelation analysis, landscape index and landscape security pattern, are also spatial analysis methods. Consideration could be given to replacing them with a specific method.**

Answer: Thank you very much for providing the feedback and suggestions again. We have carefully considered your suggestions and have changed the subtitle to "Landscape Ecological Security Analysis" in the revised version.

**2. There is a formatting error on line 174**

Answer: Thank you for pointing out our error, we have fixed the formatting error on line 174 and checked the formatting again to make sure there is no error.

**3. Coordinates are missing in Figures 2, 3, 5, and 7**

Answer: We appreciate you pointing that out. We have added latitude and longitude coordinate information to the revision and ensured that it is clearly labeled in the chart title or legend.

**Fig. 2 Land use type changes in Xishuangbanna from 1996 to 2017**

**Fig. 3 The ecological security community of the study area**

**Fig. 5 Landscape ecological safety classification map of the study area from 1996 to 2030**

**Fig. 8 Spatial clustering characteristics of landscape ecological security**

**Fig. 9 Center of gravity shift of landscape ecological security level type in Xishuangbanna from 1996 to 2030**

**-Response to Reviewer 2**

Thank you very much for your review and valuable feedback. I am very happy to hear that you think my manuscript has improved significantly in comparison to the previous version and your comments on various aspects of the paper. This is very encouraging for me.

Thank you again for your review and support. I look forward to your further guidance and suggestions.
